# Leveraging Long‐Term Ecological Research Initiatives Into the One Health Synthesis

**DOI:** 10.1002/ece3.72982

**Published:** 2026-01-20

**Authors:** Andrew G. Hope, Sam C. Speck, Zak Ratajczak

**Affiliations:** ^1^ Division of Biology Kansas State University Manhattan Kansas USA

**Keywords:** biorepository, community turnover, digitized data, emerging infectious disease, pathogen surveillance, phylogeography, specimen‐based research, woody encroachment, zoonosis

## Abstract

There is still little emphasis within One Health on building linkages between human health, changes in biodiversity, and ecosystem perturbation. We use the Great Plains, a system of substantial One Health concern, to illustrate a persistent data challenge to this issue, through the lens of small mammals, parasitic vectors of disease, and long‐term ecosystem change. We assembled specimens of small mammals to assess species turnover coupled with mitochondrial sequencing to examine intra‐specific diversity of the dominant species. We also assessed ectoparasite infection rates and related all to habitat changes. All data were retrieved from public data aggregators and combined datasets present a scenario of high potential for emergence of disease. With cessation of natural fire, watersheds that were previously grassland are now 50% woody cover following four decades of experimentation. Woody encroachment led to turnover in rodent species from *Peromyscus sonoriensis* (a grassland species) to *Peromyscus leucopus* (a woodland species), resetting the template for host‐vector‐pathogen dynamics. This was accompanied by a reduction in mammal species richness and roughly doubling in carrying capacity of high‐risk hosts and disease vectors. Hosts, vectors, and pathogens that have maintained long‐term separation may now experience increased contact as mosaics of woody encroachment bridge networks of species across the Great Plains. Knowledge of these progressive dynamics is evident through site‐intensive and long‐term sampling, and through novel data syntheses that collectively indicate heightened risk of emerging zoonotic disease throughout the region. This research promotes novel digitized data linkages between specimen time‐series, long‐term ecological research, and pathogen surveillance. We call for a “One Data” approach that emphasizes the need for building relational linkages between these vast data repositories that fuel still largely independent research priorities, but would collectively enable epidemiological advances for human health outcomes under the One Health synthesis.

## Graphical abstract

We address important ecological and evolutionary considerations for advancing a One Health perspective by drawing attention to faunal and ecosystem changes occurring through the Great Plains of North America that have strong implications for future risk of emerging pathogens including zoonoses. Our research spans multiple spatial, temporal, and taxonomic scales to address the complexity of floral and faunal turnover across the middle of the North American continent.

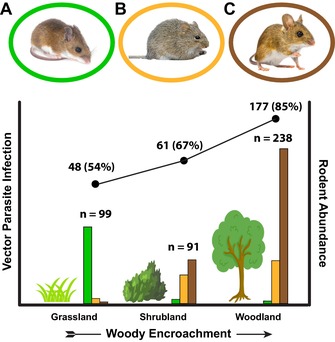

## 1 Introduction

Although not yet a household phrase, the concept of One Health is now widely recognized by health professionals as a holistic consideration of functional linkages between human health, animal health and environmental health (Destoumieux‐Garzón et al. 2018). Zoonotic diseases starting with pathogen spillover from wildlife have a long history of affecting humanity, through severely virulent outbreaks, periodic pandemics, and chronic widespread infection, including ebola, bubonic plague, influenza, HIV, and malaria (Piret and Boivin 2021). The One Health synthesis was initially developed within the purview of biomedical science and the environments of domesticated animals, but has broadened perspective to consider that our well‐being relies on improving our knowledge of biodiversity interactions at‐large, coupled with ecosystem processes. For instance, the Sars‐CoV‐2 coronavirus was linked to a spillover event between a wild mammal reservoir to humans, and subsequently from humans to multiple other species on a global scale, including both livestock and wildlife (Alluwaimi et al. 2020). Since then, One Health perspectives have increasingly considered how relationships between environmental variables such as biodiversity, ecological communities, and habitat connectivity also intersect with human population trajectories, together affecting the risk of zoonotic disease spread (Lefrançois et al. 2023). Despite advances, the One Health initiative still exhibits segregation among the major disciplines that contribute relevant data (Manlove et al. 2016). Here, we argue that much of the data and specimen resources needed to rise to this One Health challenge exist but need (1) a long‐term approach that leverages existing decadal scale ecological data along with site‐intensive, temporally deep specimen resources, and (2) development of relational linkages between data repositories from disparate fields to enable novel epidemiological exploration with the speed called for by this moment—an idea we refer to as One Data.

The complexities of host–parasite‐pathogen relationships associated with zoonoses are not new. A growing archive of research on changing dynamics of wildlife hosts, parasitic vectors, and zoonotic disease attests to recent observed shifts in the ranges of host reservoirs, pathogens, and the dynamics of exposure and infection (Daszak et al. 2000; Morand and Krasnov 2010; Hoberg and Brooks 2015; de Angeli Dutra et al. 2022). Given recent and accelerating global change, predictive models based on traits of reservoir species suggest that the geography of near‐future zoonotic disease emergence (hotspots for zoonotic risk) may not be limited to tropical latitudes or recognized biodiversity hotspots where species richness, and diversity of zoonotic pathogens is highest (Han et al. 2015). Instead, zoonoses are often associated with wide‐ranging and synanthropic reservoir species (those that maintain close associations with humans), occupying variable environments that support relatively depauperate faunas (Han et al. 2015, 2016). Several factors typify these systems: evidence of exacerbated population fluctuations of wildlife species in response to more pronounced seasonality with climate change, leading to years with high reservoir abundance (McMahon et al. 2018), more extreme weather events that impact short and long‐term population trajectories (Sergio et al. 2018), and direct linkages between loss of biodiversity and increased risk of emerging disease (Keesing and Ostfeld 2024). These are all long‐term phenomena (across decades) that are difficult to capture from short duration small‐scale studies (across seasons or up to a few years).

We focus on the Great Plains of North America in the context of a conceptual framework for how One Health practitioners and environmental scientists can mutually benefit from long‐term ecological research (LTER). These LTER initiatives include international long‐term ecological research (ILTER), long‐term agricultural research (LTAR), and a range of U.S. state and federal initiatives, such as experimental forests and agricultural research stations. LTERs fuel fundamental research that often explicitly focuses on habitat variables and ecosystem processes. This is the same arena within which relationships between hosts, parasites, and pathogens are rapidly changing. The development of accessible and relational data streams based on these experimental systems provides enormous opportunities for applied research into environmental health, maintenance of biodiversity, and human health, pillars of the One Health synthesis.

The Great Plains are one of Earth's largest grassland ecosystems, which in many respects epitomize an emerging high‐risk system for zoonoses (Han et al. 2015; Keesing and Ostfeld 2024). Global grasslands are among the most human‐altered ecosystems on Earth (Hoekstra et al. 2005; Scholtz and Twidwell 2022). The Great Plains are located at the crossroads of faunal communities distributed across multiple distinct ecosystems organized around strong climate gradients that drive community turnover across both latitude and longitude, and yet support relatively low faunal diversity and endemism (Jenkins et al. 2015). This region also exhibits high environmental seasonality coupled with extensive and progressive human‐induced land‐cover change, and associated declines in native biodiversity (Ratajczak et al. 2022).

For a finer scale perspective, we narrow our focus to the U.S. state of Kansas. At the geographic center of North America, we argue that Kansas reflects a pivot point for both biotic and abiotic components of Great Plains environments that spans the scope of these variables (e.g., Burke et al. 1991). Like many other developed and grazed land areas in Africa, Asia, Australia, Europe, and South America, Kansas and the Great Plains at large have a high percentage of land tenure under the control of private landowners. Continuing land‐use intensification in increasingly fragmented landscapes is expanding the potential for interactions with disease vectors (Diuk‐Wasser et al. 2021; Ortiz et al. 2021). Private land tenure also means that affecting meaningful changes will require demonstrating overwhelming evidence of risks to humans with inaction and broad benefits of supporting mitigation, because land‐use decisions that reduce zoonotic disease exposure often cut against immediate economic benefits (Vanwambeke et al. 2024).

**FIGURE 1:**
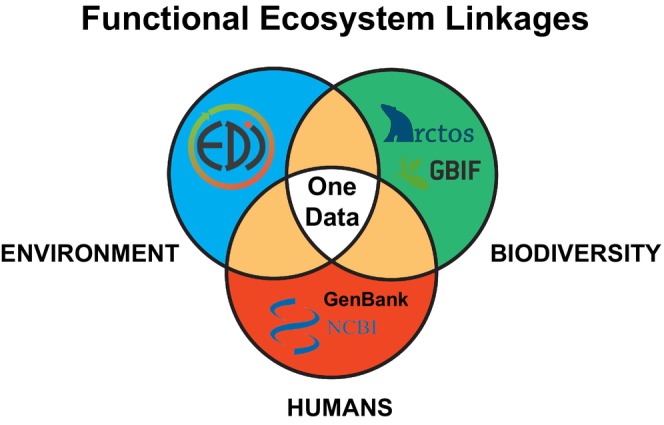
A One Data perspective on the One Health framework. This is a conceptual framework where enabling rigorous research on One Health will require relational linkages between digitized data‐streams relevant to Biodiversity (such as the Arctos multi‐institutional museum database or the Global Biodiversity Information Facility that aggregates archived specimen data), Environments (such as the Environmental Data Initiative that aggregates ecological data from long‐term research ventures), and Humans (including pathogen genomic data from the National Center for Biotechnology Information, including GenBank).

Using information gathered across Kansas and the Great Plains, we demonstrate that continued development of targeted linkages between diverse data‐streams across both life sciences and geophysical sciences can positively impact One Health outcomes—an idea we term “One Data” (Figure 1). One Data is the concept that tackling One Health in a meaningful way will require currently siloed data‐streams and expertise to come together and achieve synergistic inference that is currently lacking.

### 1.1 The Challenge for One Data Linkages

Evolutionary, ecological, and biomedical sciences are all increasingly understood as essential components towards achieving positive One Health outcomes yet are traditionally independent disciplines that lack clear data connections (e.g., Manlove et al. 2016). This paper demonstrates the potential for exploring zoonotic disease by combining datasets that are not yet functionally relational for the broader scientific community. For this we use systematic and targeted sampling of small mammal specimens for evolutionary and host–parasite analysis that explicitly parallels the rigor of associated long‐term ecological data series from ecosystem sciences (e.g., Hope et al. 2018). Developing such synergy more broadly would allow exploration not only of host evolutionary legacies but temporal trends in parasite and pathogen diversity through genomic surveillance based on time series of modern preservations, effectively bridging the gap across disciplines.

Still, the hurdle is developing digital connections, so‐called relational linkages, that provide ready access to associated data collected through solo or trans‐disciplinary efforts. These can be reciprocal hyperlinks between a museum specimen, an environmental dataset, and a pathogen genetic sequence (Figure 1). All of these are held in multiple independent databases, but are rarely explicitly linked, meaning that the existing relationships between them are not easily realized by potential data users. For instance, specimen data accessible through the Integrated Digitized Biocollections (iDigBio), ecological data held in the Environmental Data Initiative (EDI), and host/pathogen genetic data held within GenBank (all as examples of respective data repositories developed from North America) are still not readily relational in most cases. This is despite each repository being a leading initiative towards mobilizing digitized data.

The very present global need for One Health, a framework that increasingly relies on all three data streams, presents a strong opportunity to catalyze such linkages. Currently, the leading example of robust relational linkages within biodiversity sciences hinges on the existence of vouchered host specimens held in digitized collection databases (e.g., arctos.database.org; Cook and Light 2019). This highlights another intrinsic value of physical specimens that, more than simply recorded data points (e.g., field measurements), offer tangible source material that can be revisited and re‐used repeatedly as One Health investigations develop more intricate questions through relational pathways (e.g., Thompson et al. 2021).

### 1.2 Using the Great Plains to Understand Extrinsic Changes to Environmental “Health”

Before western settlement of the Great Plains in the early 1800s, expansive grasslands stretched from northern Mexico to central Canada and were maintained by relatively intact and stable consumer guilds, fire regimes, and climatic gradients (Axelrod 1985; Samson et al. 2004). In the past 150 years, local land conversion of the Great Plains region, wholesale declines in native wildlife (Flores 2017), and global anthropogenic forcing have transformed these grasslands to an increasingly fragmented mosaic of remnant habitats, agriculture, urban zones, and woodland (Samson et al. 2004; Scholtz and Twidwell 2022). Impacts on global climate systems include increases in the intensity and severity of extreme events, such as droughts that are more severe and longer than in recent centuries (Cook et al. 2015).

The stability of wildlife has also been progressively altered. The Great Plains grasslands historically divided forested ecosystems of western and eastern North America, while also maintaining a strong filter for dispersal of species (e.g., Reding et al. 2021) and pathogens (e.g., Alkishe et al. 2021). Steep climate gradients also played an important role, with large changes in precipitation from east to west, and in temperature from north to south. Small mammal communities of Great Plains grasslands transition in both diversity and composition from western arid grasslands (higher richness and western origin species) to eastern mesic grasslands (lower richness and greater influence of plains endemics and eastern origin species; Jenkins et al. 2015). Similarly, components of mammalian communities associated with north‐temperate grassland and boreal zones dominate in the northern plains, and species typical of sub‐tropics and arid chaparral push into the southern plains (Armstrong et al. 1986; Hope et al. 2026). This turnover of communities across the middle of the continent translates to relatively high total diversity across this vast region, but relatively low local diversity or endemism (Jenkins et al. 2015), with community structure in a given area maintained by the consistency of strong environmental gradients, at least through ecological timeframes (Johnsgard 1978).

But environmental changes are accelerating. Warming global climate and rising atmospheric CO_2_ have shifted the Great Plains climatic envelope north by over 500 km in the last 50 years (Roberts et al. 2019). Similarly, the transition zone between arid western grasslands and eastern mesic grasslands has shifted east from roughly the 100th meridian to the 98th meridian longitude in the same time frame (Seager et al. 2018). With these atmospheric shifts are concomitant changes in environmental drivers. Cessation of natural wildfire regimes has substantially contributed to westward woody encroachment by eastern woodland flora (Ratajczak et al. 2016; Archer et al. 2017). Invasive species are spreading throughout the Great Plains, resulting in patchier prairies, often confined to more upland and marginal soils that remain drier and less productive (Van Auken 2009). At the broadest scale, remaining unfragmented tracts of Great Plains grasslands are often relegated to the arid short‐grass and mixed‐grass regions of the western and northern Plains (Samson et al. 2004). This means that locally distinct lineages (evolutionarily discrete intra‐specific units) and ecotypes (adaptive population units) of biota that occur in more mesic regions of the eastern Plains (east of the 100th meridian) are experiencing highest levels of change (Galliart et al. 2019; Hope et al. 2026).

### 1.3 Joint Consideration of Evolutionary and Ecological Data Streams

Because native grasslands globally are experiencing massive reduction through human action (Scholtz and Twidwell 2022), they are also the focus of extensive and often multi‐decadal ecosystem studies and provide opportunities to repurpose data towards emerging considerations, including One Health. In the Great Plains, long‐term data collection through broad‐scale monitoring efforts allows us, at least in part, to summarize environmental and biodiversity dynamics (Schmidt et al. 2021) and quantify change through landscape‐scale experimental initiatives (e.g., Ratajczak et al. 2014; Bruckerhoff et al. 2020; Hope et al. 2021). Data on trends of small mammals across this region span methods that include spatially broad, site‐intensive, and temporally deep specimen sampling. However, rigorous biodiversity archives are much less consistent across the global stage, often given perceived ethical conflict between biodiversity conservation and science‐based specimen collection (McLean et al. 2016; Nachman et al. 2023).

Natural history collections and biorepositories are archives of physical specimens that record the existence of species at a given place and time and represent a permanent record of biodiversity from which we can assess change thanks to persistent sampling effort across decadal timeframes (for illustrated reviews, see figures in Dunnum et al. 2017; Miller et al. 2020). Continuing series of specimens through time, including progressive changes in methods of collection and preservation of specimen materials, can greatly extend the value of these resources towards understanding disease dynamics, community turnover, evolutionary trends, and many other emerging applications (Galbreath et al. 2019; Paul et al. 2025). Whole‐specimen resources allow for inter‐disciplinary collaborative research and educational initiatives to build knowledge of the connections between extrinsic environmental variables and the intrinsic traits of species that collectively govern the likelihood of disease emergence and transmission (Cook et al. 2014; Colella et al. 2021).

In recent years, shifting priorities in conservation biology, biomedical science, and precedence of national funding (in the U.S. and elsewhere) have led to major advances in data digitization and dissemination, allowing for aggregated online information on species distributions and traits based on specimen resources (Hedrick et al. 2020; McLean et al. 2025). This change reflects an accelerating demand for use of specimens in multi‐disciplinary science, including One Health (Thompson et al. 2021). Kansas, and the Great Plains in general, have a long history of mammal biodiversity research through field sampling and curation of specimen resources (Flores 2022), and these efforts are recorded through specimen archives housed in museums across North America and available through digitized specimen catalogs (e.g., the Global Biodiversity Information Facility [GBIF] and Integrative Digitized Biocollections [iDigBio]).

In addition to a region‐wide specimen record of mammal occurrence data, certain local sites have emphasized more site‐intensive efforts. In 1980, the U.S. National Science Foundation commenced funding for the Long‐Term Ecological Research Network with an overriding emphasis on experimental data collection to understand processes of ecosystem change. The Konza LTER site in northeast Kansas, one of three LTER sites that have explicitly focused on North American grasslands, is also one of the original landscape‐scale experimental stations within this network and has collected environmental data, and floral and faunal data for over 40 years (Knapp et al. 1998). Included in the research priorities is understanding how populations of consumers influence ecosystem state and how drivers of change impact consumers.

Konza is one of only two U.S. LTER sites (along with the Sevilleta LTER in New Mexico) that regularly combine long‐term small mammal monitoring data with site‐intensive and temporally deep specimen sampling, with an explicit goal of enabling scientific inquiry into changes in intrinsic traits of small mammals as related to experimental manipulation of ecosystems (e.g., Hope et al. 2021). This includes their genetic legacies (through collection of genomic resources), trophic relationships (through stable isotopic analysis of multiple tissue types), parasite biodiversity and co‐evolution (through preservation of ecto‐ and endoparasites), and disease dynamics (through host and parasite pathogen surveillance).

Data sampled from both landcover change and small mammal reservoirs can yield surprises. For instance, we recently found that reintroducing frequent fires to an encroached area reduced shrub height by half and allowed much of the grass layer to recover (Collins et al. 2021). However, woodland‐associated species still dominated the small mammal community (Hope et al. 2021). If we had relied on vegetation alone, we would have predicted a small mammal community dominated by grassland species, missing a strong legacy effect of the remaining woody vegetation. As such, an investment like the Konza LTER is uniquely poised to fuel One Health perspectives by coordinating data streams that include both robust time‐series of whole‐specimen resources and rigorous long‐term ecological data from a landscape‐scale experimental system.

### 1.4 Intrinsic Traits of Focal Reservoir Species

A decade ago, the risks of emerging zoonotic disease through development of new host‐pathogen relationships between small mammals and their parasites (considering micro‐parasitic pathogens) were summarized on a global scale using trait‐based predictive modeling (Han et al. 2015). The central Great Plains of Kansas and Nebraska were highlighted as one potential hotspot for the development of emerging zoonotic disease as a function of the geography and associated environments of this region and based on multiple life history traits of resident small mammal faunas. Other predicted regions, also strikingly coincident with the distribution of northern hemisphere grasslands, include central Asia (Kazakhstan) and eastern Asia (northern China). The Great Plains offers a tractable opportunity to develop rigorous epidemiological investigations based on existing and continuing data streams that might inform us more broadly of risks associated with other similar global hotspots.

Critically, three of the most common small mammal species associated with the central Great Plains are within the top 15 reservoirs for known zoonoses globally, out of over 2200 small mammal species included in the predictive models (Han et al. 2015), and these three species comprise our focal taxa. More generally, the vast majority of all zoonoses are associated with wild mammals, and there is a strong and direct positive relationship between diversity within mammalian orders and the diversity of detected pathogens (Han et al. 2016). The specific focus on small mammals, and rodents in particular, for assessing zoonotic disease risk is therefore warranted, given that rodents constitute roughly 40% of all mammals.

### 1.5 Synthesis

Here we provide a One Data synthesis to demonstrate the potential for extrinsic changes across Great Plains ecosystems to amplify the risk of zoonotic disease through concomitant responses of intrinsic components of biodiversity as they respond to long‐term perturbation. We present a synanthropic system where native grassland habitats are progressively changing to woody habitats more typical of further east and south within North America, leading to cascading changes in small mammal communities, prevalence of ectoparasitic vectors of disease, and relative carrying capacities of both. This region has the hallmarks of a “hotspot of risk” of emerging disease, despite not being in the tropics or a biodiversity hotspot. We explain the rationale for how these dynamics interplay by relating modeled predictions for just such a scenario (Han et al. 2015) to a real‐life system of change that urgently warrants further scientific investigation. Although we present here elements of new information (e.g., re‐analyzes, extensions of existing datasets), we emphasize through synthesis of existing long‐term data streams the critical need for building One Data linkages. The hope is that future digital integration of previously independent research initiatives will enable practitioners to refine and amplify scientific advances towards enhancing human health.

## 2 Methods

### 2.1 Focal Small Mammals

**FIGURE 2:**
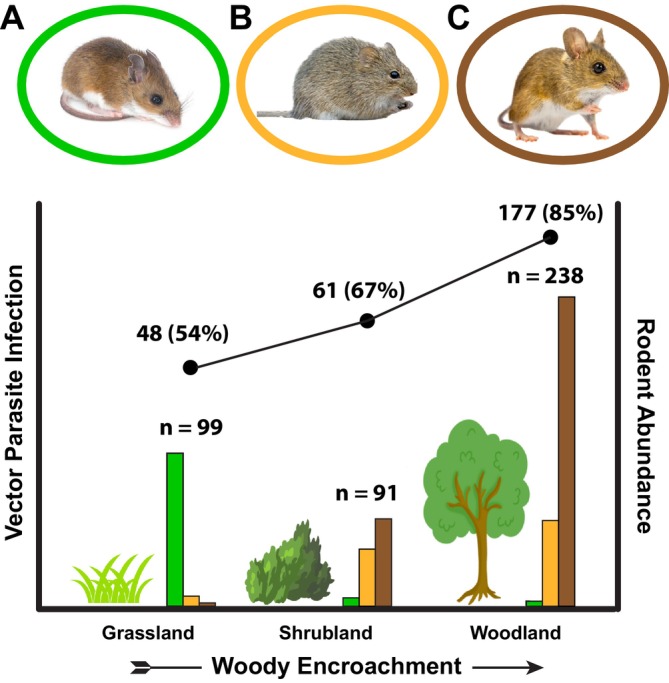
Top: Rodents relevant to Great Plains zoonotic potential, including (A): Western deer mouse (*Peromyscus sonoriensis*), (B): Hispid cotton rat ( *Sigmodon hispidus* ), and (C): White‐footed mouse ( *Peromyscus leucopus* ). These species are predominantly associated with grassland, shrubland, and woodland, respectively. Bottom: Biodiversity dynamics reflecting data from the Konza Long‐term Ecological Research site. The curve illustrates an increase in parasitic vector infection of sampled rodents from grassland to woodland based on screening from the last 5 years (number and percentage of total catch infected). The histogram illustrates abundance of the three focal rodent species from each sampled habitat in the last 5 years combined, where values represent totals of all three species, by habitat, reflecting an associated increase in carrying capacity with woody encroachment.

Species of high zoonotic concern considered in the current synthesis include the western deer mouse (*Peromyscus sonoriensis*; previously *P. maniculatus* ), the white‐footed mouse ( *P. leucopus* ), and the hispid cotton rat ( *Sigmodon hispidus* ; Figure 2). Collectively, these species are known to carry zoonotic pathogens (often vector‐borne) including viruses (e.g., Hantavirus, Powassan), bacteria (e.g., *Borrelia*, *Ehrlichia*, *Yersinia*), and protists (e.g., *Schistosoma*, *Trypanosoma*) (Barbour 2017; Rochlin and Toledo 2020).

Deer mice, including the western deer mouse, are wide‐ranging across North America and can be found in virtually all terrestrial habitats. These mice are perhaps the nearest to a “model” for scientific inquiry among wildlife species (Bedford and Hoekstra 2015). Despite range‐wide complexity, deer mice across the central Great Plains constitute a single intra‐specific lineage (Dragoo et al. 2006; Hope et al. 2026). They are grassland obligate mammals rarely detected in even modestly shrubby habitats (Pergams and Nyberg 2001). They respond positively to frequent grassland fires and are the numerically dominant small mammal species throughout most native prairie regions that experience periodic fire (Kaufman et al. 1988). Carrying capacity of rodents in these open habitats is characteristically low (Hope 2023; Rowland‐Schaefer et al. 2024). Although deer mice collectively harbor among the highest known diversity of zoonoses of all small mammals across their range (Barbour 2017), low intra‐specific genetic diversity within the Great Plains (Hope et al. 2026) likely reflects lower resident pathogen diversity within grassland deer mice than within the broader species complex.

White‐footed mice are a woody‐associated species of eastern and southern North America. The distribution of this species is expanding through the Great Plains associated with encroachment of woody habitats, and densities of white‐footed mice within newly invaded woody habitats rapidly exceed that of congeneric deer mice within pre‐encroachment communities (Clark et al. 1987; Figure 2). Given that woody encroachment is the predominant habitat trend being experienced across the study region, white‐footed mice are our primary focus for changing host dynamics.

Hispid cotton rats are a southern sub‐tropical species associated with a heterogeneous mix of habitat types. This species epitomizes an extreme boom‐bust life history with a brief lifespan (six months) and ability to respond rapidly to interannual environmental variability (Odum 1955). These traits are positively associated with disease potential, and like *Peromyscus*, hispid cotton rats are also a reservoir for multiple zoonoses (Mills et al. 2010). This species is rapidly expanding its range northward as populations experience warming climate trends. In low‐density years, this species often becomes scarce to the point of being undetectable using standard trapping methods, potentially also exacerbating relationships between co‐distributed pools of hosts, vectors, and pathogens as communities temporarily restructure (Ecke et al. 2017). The hispid cotton rat is a potential wildcard in this dynamic environment, influencing amplification or dilution dynamics across very short timespans, and with little ability to predict or mitigate short‐term outcomes (Levi et al. 2016; Luis et al. 2018).

### 2.2 Data Acquisition and Synthesis

We compiled both statewide and local site data from small mammals to reveal community turnover, as related to quantifiable anthropogenic habitat change, changes in small mammal populations across spatial scales of analysis, and related changes in both host genetic legacies and vector infection prevalence, all relevant to altered risks of zoonoses. We queried digitized museum catalogs including GBIF (GBIF.org 2024) and Arctos (arctos.database.museum) for focal mammal species distributed across Kansas to assess relative abundance as represented by archived voucher specimen resources collected between 1980 and 2020 (40 years), and to reflect turnover in numerical dominance of potential hyper‐reservoirs of zoonoses. Analysis of statewide specimen sampling included five‐year sliding average gross abundance of specimens from each focal species.

We then summarized data from small mammal transects, long‐term vegetation data, and aerial imagery surveyed across the Konza LTER, also between 1980 and 2020. Small mammal sampling sites varied by fire return intervals, including an annual, a four‐year, and a 20‐year burn treatment that have been applied consistently throughout the data collection interval, resulting in a clear relationship between burn frequency and levels of woody encroachment. The small mammal data from these sites, downloaded from the Environmental Data Initiative (EDI; Kaufman 2021; Hope 2023), includes capture‐mark‐recapture records of relative abundance from linear live‐trapping transects annually. Voucher specimen collection occurred on half of all trapping transects between 2015 and 2020, providing tissue resources for genetic analysis, endoparasitic helminths, and ectoparasitic vectors, all retrieved from mammalian hosts (Hope 2023). We procured tissue loans from these resources based on digitized specimen records from the Arctos multi‐institutional database, parsing the diversity of evolutionary complexity and assessing relative prevalence of ectoparasite infection within focal small mammals across fire treatments.

For each mammal species within each burn treatment we developed general additive models of abundance through time as related to changes in land‐cover summarized from long‐term plant composition transect data similar to methods of Collins et al. (2021) and Ratajczak et al. (2022). The vegetation data come from a network of 40 10 m^2^ plots (per fire treatment) (from EDI; Hartnett et al. 2024), which we used to develop a general additive model for each combination of burn frequency and plant functional type (extended from Collins et al. 2021; Ratajczak et al. 2022). We also include aerial imagery from the USDA NAIP program (Maxwell et al. 2017), NSF NEON (NEON 2022), and locally derived land‐cover classes created with machine learning (Noble and Ratajczak 2025). The aerial data give wall‐to‐wall cover and arrangement of woody vegetation expanding into the grasslands of each treatment.

Ectoparasite data consisted of relative incidence of infection of small mammals, including ticks (and other mites), fleas, lice, and botfly larvae. For white‐footed mice, we compiled genetic data based on up to 1143 base pairs of the mitochondrial cytochrome b gene, including available sequences downloaded from the NCBI GenBank, plus sequences from selected samples from the Konza LTER site and elsewhere across the Great Plains (Supporting Information). Phylogeny construction using Bayesian statistics was performed to identify robust monophyletic intra‐specific lineages, which we related to geographic distribution by plotting georeferenced locality records, all following methods of Herrera (2021).

## 3 Results

### 3.1 Descriptive Epidemiology of Great Plains Host‐Environment Relationships

**FIGURE 3:**
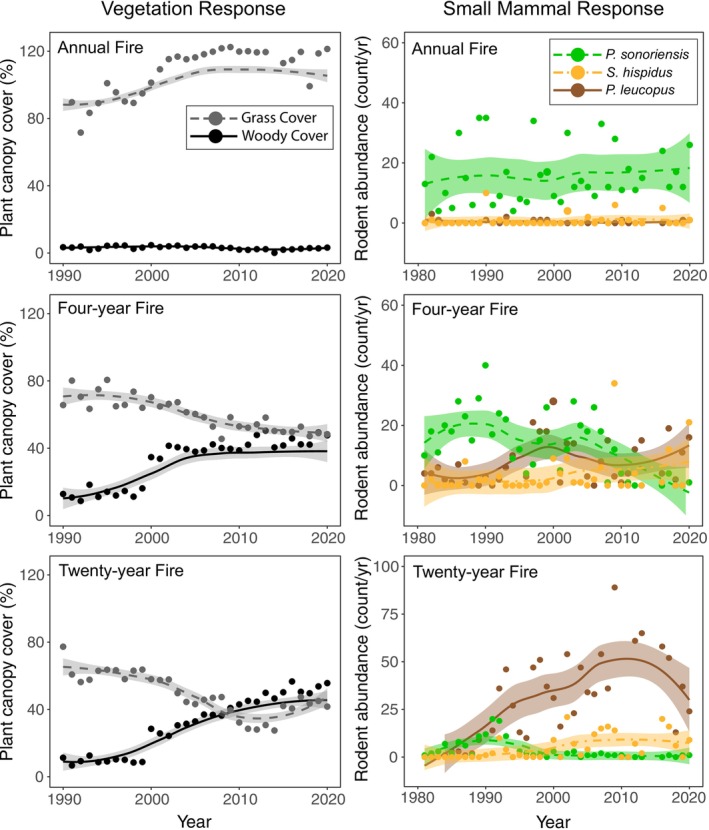
Plots illustrating long‐term changes in prevalence of both vegetation types (left) and focal small mammal species (right) in response to ecosystem state as a consequence of different prescribed fire regimes. Annual fire maintains native grassland and suppresses occurrence of woody vegetation and woodland‐associated small mammals. Four‐year fire leads to a lagged transition in vegetation to woody shrubs and an associated slow turnover in dominant small mammal species. Twenty‐year fire leads to a lagged transition in vegetation towards a woodland state of both shrubs and overstory trees (shown in detail in Figure 4) and a rapid turnover in dominant small mammals accompanied by a substantial increase in carrying capacity through time.

**FIGURE 4:**
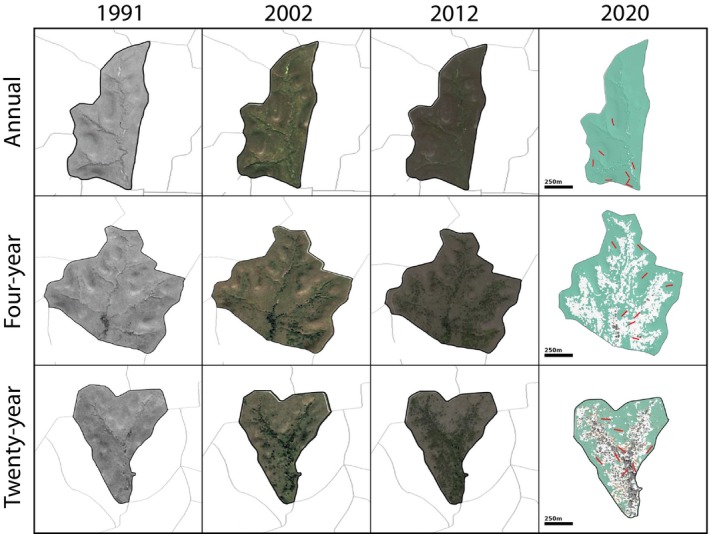
Images through time illustrating vegetational changes in treatment areas included in this study including Top: Annual fire, Middle: Four‐year fire, and Bottom: Twenty‐year fire. Images were retrieved from Google Earth for years 1991, 2002, and 2012. Images for year 2020 were generated using remote aerial sensing coupled with ground‐proofing to illustrate grass, shrubs, and trees in finer detail. Included are red lines showing long‐term vegetation sampling transects.

**FIGURE 5:**
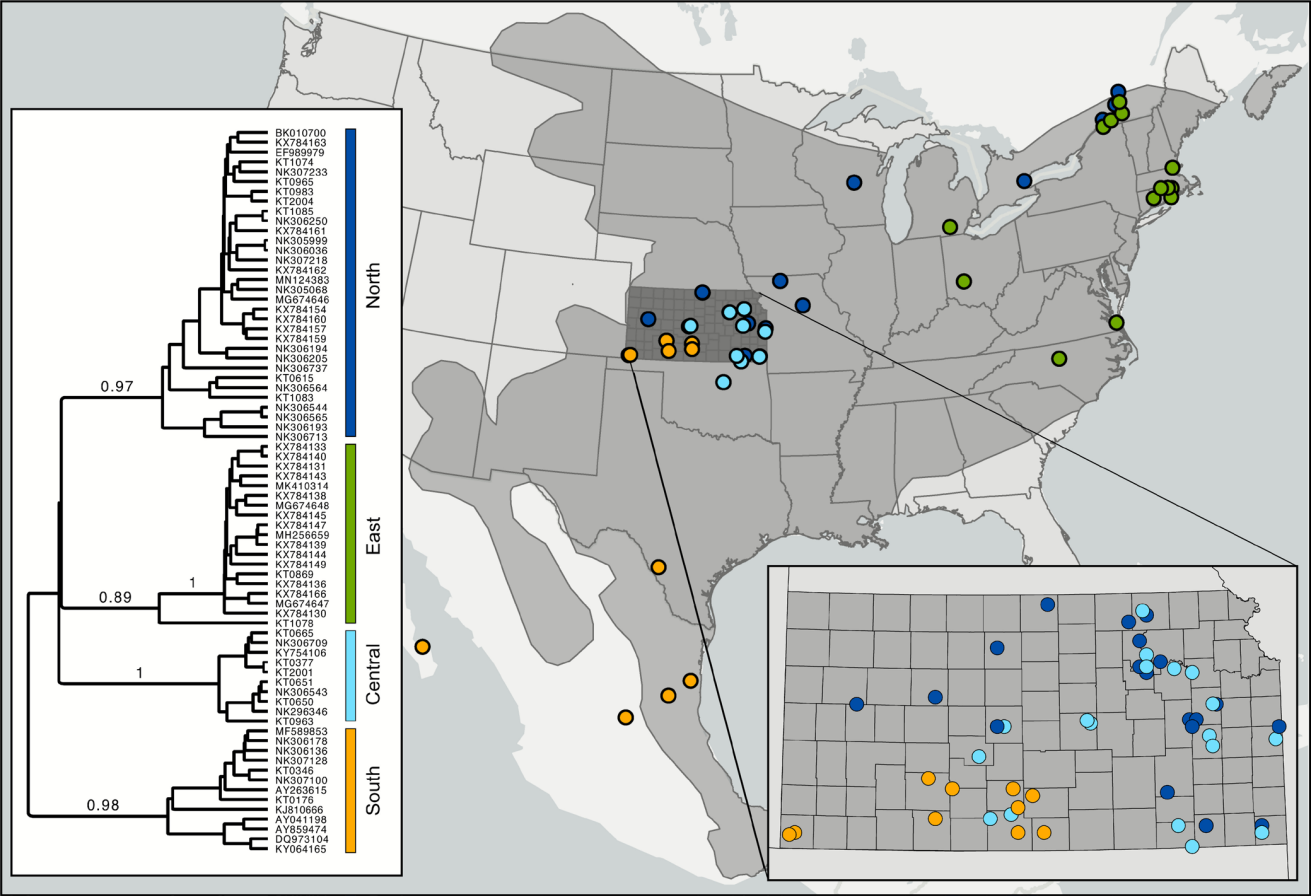
Phylogeographic relationships among lineages of white‐footed mice, colored according to geographic regionalization. The map of North America illustrates the rangewide distribution (gray shading) with colored data points showing the samples included in phylogenetic analysis of the mtDNA cytochrome b gene sequences. The Bayesian phylogeny (left) shows four well‐supported lineages distributed through east, north, south, and central portions of the range. The inset map shows lineage representation within Kansas where six sampled localities support individuals from multiple mitochondrial lineages, including the Konza LTER site.

We have summarized results from close to a half‐century of long‐term ecological and specimen data collection on changes in habitats, climate, and small mammal populations through the central Great Plains, towards greater understanding of zoonotic disease risk. Given the application of a multi‐faceted approach based on One Data linkages, the following observations are evident from our combined data streams:

1. Regular fires helped to maintain native grasslands. Annual burns remained as open native grassland; four‐year burns changed slowly at first but eventually became dominated by woody shrubs, and 20‐year burns are approaching a woodland state following decades of almost complete fire suppression (Figures 3 and 4).
2. Both locally (Figures 2 and 3) and regionally (Figure S1), where habitats have changed, small mammal communities have experienced turnover in dominant species, and the abundance of the most common native grassland small mammal (deer mouse; *Peromyscus sonoriensis*) has declined while the abundance of the most common woodland species (white‐footed mouse; *Peromyscus leucopus* ) has increased. This is coupled with a change in carrying capacity, where the number of captures of deer mice in grasslands was low but the numbers of white‐footed mice in woody habitats was about twice as high on average (Figures 2 and 3).
3. Infection rates by ectoparasites (mostly flea and tick vectors of disease) were very high from rodents in recently encroached woody habitats but substantially lower from rodents in remaining native grassland (Figure 2).
4. Intra‐specific lineage diversity of the declining deer mouse was low, whereas lineage diversity of the expanding white‐footed mouse was higher, translating to increasing complexity of lineage‐specific or inter‐lineage host‐pathogen dynamics through time (Figure 5). In Kansas, three lineages converged, with multiple localities that support genetic haplotypes associated with multiple lineages (Figure 5; Hope et al. 2026). The high lineage diversity of these mice through the Great Plains likely amplifies zoonotic disease potential (Ostfeld and Keesing 2012; Fountain‐Jones et al. 2018).

## 4 Discussion

### 4.1 Inference of Disease Risk Based on Cascading Biotic Changes

The available data provide compelling correlative evidence for biotic state change across ecosystems over the course of a single human lifetime (Briggs et al. 2005; Nippert et al. 2021). We acknowledge that cause and effect within this system as related to risks of emerging zoonotic disease are still not firmly established. A primary strength of this synthesis is in providing a methodological model to inform future pathogen explorations based on this system, and for investigating other similar at‐risk systems globally. Our analyzes reveal relationships that leverage multiple existing comparative datasets, with consistency in ongoing and future data collection, and as such offer opportunity for more targeted epidemiological investigation based on actively expanding specimen archives. This is an instance where we cannot make exact predictions of when and what types of pathogen evolution will occur; however, all lines of evidence point towards greater risk of novel zoonoses.

We can interpret our observations as follows: Native grasslands through the Great Plains are experiencing woody encroachment, causing fragmentation and loss of grassland habitat (Samson et al. 2004). These fragmented mosaics create a large surface area between grassland and woody vegetation, where taxa from each habitat can come into contact (Figure 4). Declining area of grassland reduces abundance of the dominant deer mouse, which is evident at landscape and state‐wide scales. Across the region a woodland species of rodent, the white‐footed mouse became the dominant small mammal species recently in the mid‐2000's (Figure S1). This process of turnover has not been instantaneous (Figure 3), with a lag period of nearly a decade in both the vegetation and mammal community, highlighting the importance of sustained site‐intensive data collection. This transitional phase through the process of woody encroachment also reflects high potential for mixing between host small mammals, vectors, and pathogens. At this point, as much as half of the central Great Plains region is transitional with respect to dominant native habitats (Ratajczak et al. 2016)—a critical time for developing data resources to capture the impacts of these processes.

A decline in native grassland indicator species, such as the deer mouse, signals broad regional changes in biodiversity (Pergams and Nyberg 2001). We are also seeing substantial declines in grassland birds across the Great Plains (e.g., Bernath‐Plaisted et al. 2023), heralding consideration of the conservation and associated health implications of progressive loss of the deer mouse and other grassland species (Lindenmayer et al. 2011). Infection of deer mice by ectoparasitic vectors of disease based on our data was low. Similarly, carrying capacity for these rodents within grasslands was consistently low compared to densities of rodents in woody encroached areas. This combination of low vector and low reservoir abundance collectively reflects low risk of disease spread within the native grassland habitats that historically were much more widespread (Han et al. 2016; Keesing and Ostfeld 2024).

It is therefore possible that historic fire regimes not only maintained grassland mammal communities but also limited disease potential, although this has had little exploration from a functional perspective (Axelrod 1985). In ecosystems where fires are disruptive, fire tends to have an opposite effect and may increase the likelihood of disease (Albery et al. 2021). However, grassland ecosystem structure and function are often positively maintained by fire (Briggs et al. 2005; Bond 2008). In grassland systems, fire can directly reduce free‐living vector populations and free‐living life‐stages of potentially pathogenic parasites (reviewed in Scasta 2015). In areas that remain grass‐dominated, frequent fire still has a positive impact on the incidence of deer mice (Rowland‐Schaefer et al. 2024). Therefore, short‐term cessation of fire may lead to direct increases in parasite loads and declines of native grassland host species. Longer‐term fire suppression results in woody encroachment, a progressive increase in carrying capacity of woodland mice as hosts of disease, and substantial further increases in parasite vectors (Figure 2).

Woody encroachment is a pervasive issue in grassland systems globally and in the Great Plains is progressively advancing both northward and westward (Engle et al. 2008). Decoupling the role of encroachment versus other related variables towards disease risks is difficult. Similar geographic shifts are occurring with rising average temperatures and expanding ranges of multiple mammal species, tick species, and known zoonoses, especially those transmitted by ticks (Alkishe et al. 2021). Our data support an almost complete turnover in dominant small mammal species within woody habitats, but also a decline in diversity, with only three small mammal species constituting over 95% of captures in woody areas in recent years and a doubling in their relative abundance. And because our experimental management units are in proximity, we have evidence of localized encroachment as a direct driver of changes in the small mammal community (Figures 3 and 4). Declines in mammal diversity with woody encroachment have been documented previously, with negative implications for the integrity of native grassland communities more broadly (Clark et al. 1987; Horncastle et al. 2005). Low diversity and high densities of potential reservoirs coupled with high infection rates by vectors all increase disease risk (Han et al. 2016; Keesing and Ostfeld 2024).

Evolutionary complexity within a system can also influence propensity for disease spread and development of novel host‐pathogen relationships (Ostfeld and Keesing 2012; Ecke et al. 2022). The specificity of both vectors and pathogens to mammal reservoirs can be strongly co‐evolved, may result from parallel co‐diversification through neutral evolutionary processes (e.g., drift within small, isolated populations), or may be weak, where multiple hosts prove competent to infection by vector and/or pathogen (Hoberg and Brooks 2015). We must strive towards greater understanding of host–parasite evolutionary relationships, as currently, little information from natural communities is available to guide treatment of ongoing ecosystem state change, when considering One Health (Poulin et al. 2024).

Recent evidence from phylogeographic studies of small mammal hosts posits that regions of overlap between closely related host lineages may increase opportunity for co‐evolved parasites and pathogens to experience secondary contact following long‐term isolation and differentiation. This leads to potentially acute evolutionary outcomes that might include host hybridization, switching of hosts by parasites, or pathogen genomic reassortment leading to new strains of disease (Dragoo et al. 2006; Liphardt et al. 2020). The potential for amplification of emerging disease risk within the central Great Plains is high, given the co‐distribution of multiple intra‐specific lineages of white‐footed mouse (at least six known collection localities supporting multiple lineages, Figure 5) coupled with an accelerating increase in local densities and expanding distributions; a trend that warrants epidemiological attention (Luis et al. 2018).

We acknowledge that the single mitochondrial gene used to identify intra‐specific functional units of analysis is likely only a loose approximation of the evolutionary relationships among populations. But the lineages within white‐footed mice are both geographically and ecologically diagnosable (Walters et al. 2024). Further, distinct reciprocal monophyly based on this dataset reflects previous episodes of allopatry and differentiation as opposed to simple isolation by distance, with potential for similar differentiation among both hosts and pathogens that will require future comparative phylogenomic assessment (McGaughran et al. 2022).

Beyond the intricacies of co‐evolution among pairs of hosts and pathogens, the Great Plains generally are a hub for potentially long‐term interactions between western and eastern faunas of North America (Herrera 2021). The strong longitudinal precipitation gradient across the 100th meridian has been a constant ecological filter and transition zone between western and eastern communities across evolutionary timeframes, and minimally through the last glacial cycle. Evidence from across multiple small mammal communities with different evolutionary origins highlights long‐term associations between west and east through the Great Plains, with a persistent transition from grassland to eastern woodland through this region, that shifted southward during glacial phase(s) and northward during interglacial warm periods such as at present (Herrera 2021). A breakdown of native habitats through human‐induced change is in effect making this biodiversity filter leaky, and progressively mosaic‐like habitats may exponentially increase edge effect interactions between otherwise discrete communities, resulting in increased potential for emerging disease (Diuk‐Wasser et al. 2021; Goethert and Telford III 2022).

### 4.2 Future One Health Actions for the Great Plains and Other Grassland Systems

We are now experiencing increasing incidence of zoonotic disease transmission across the Great Plains (Han et al. 2016; Eisen et al. 2017). There are both ultimate and proximate considerations for emerging disease within this region. Here we have tried to summarize proximate issues through species interactions, contemporary evolution, and fine‐scale ecological change across inter‐annual timeframes. However, the sum effects of these risks will not be fully understood without a concerted effort to expand the scope of practitioners within One Health investigations, and through coordinated collection and analysis of novel combinations of diverse data.

We suggest that linking and incorporating ecological data from long‐term research initiatives with specimen‐based trait data has disproportionate potential to offset a knowledge gap within One Health. This would include focusing long‐term data collection on components of biodiversity that are traditionally not a variable of interest for understanding long‐term ecological change, but that are direct contributors or antagonists to pathogen proliferation. For example, targeting surrogate biodiversity such as macroparasites (helminths) that may themselves be pathogenic would minimally expand our purview of intricate biodiversity dynamics through their complex lifecycles that bind together the dynamics of disparate phyla of intermediate hosts (Rowan et al. 2023). Parasites respond directly to ecosystem drivers such as fire and indirectly through disruption or promotion of intermediate hosts. For instance, decline in northern bobwhite quail has been linked to nematode infection that requires a prominent grasshopper as an intermediate host (Kistler et al. 2016). In this scenario, grasshoppers and the fire that could conceivably disrupt parasite lifecycles are both known drivers of ecosystem state (Welti et al. 2019). A growing body of literature discusses the importance of dilution effects to bolster One Health outcomes, where higher diversity in a system tends to decrease the negative impacts of a subset of harmful species (Keesing and Ostfeld 2021). Macroparasite diversity has been implicated as a critical variable influencing potential for microparasitic infection (Johnson and Hoverman 2012; Bordes et al. 2015). It is also possible that ecological theories including dilution effects may play an informative role in the macroecology of ecosystem change or stability that would benefit long‐term experimental systems (e.g., Tilman et al. 2006).

A critical component of future research efforts will be establishing pathogen surveillance associated with long‐term ecological research, using genomic approaches (Gardy and Loman 2018; Colella et al. 2023). Genomic methods will enable detection of different pathogens through generation of strain‐ or lineage‐level genetic sequence datasets from both hosts and their vectors, across multi‐year timeframes, as opposed to surveillance of pathogen exposure through antibody testing. From these data, we might better track changes in pathogen‐reservoir relationships through time, genomic legacies of co‐evolutionary relationships of lineages, and emergence of newly evolved variants.

Reaching this goal demands a multi‐faceted approach, such as building relational data systems across data sources from different scientific disciplines that allow disparate sources to smoothly “talk” to each other (e.g., as outlined for museum collections data in Cook and Light 2019). Functionally, this will necessitate streamlining data carpentry tools and incorporating this toolset into training programs of study at the undergraduate and graduate levels. For training, many programs in biomedical science, evolutionary biology, molecular biology, ecology, and geography rightfully require training in statistics. But similar emphasis on data carpentry remains rare, leading to common perceptions that skills such as working with relational databases and “big data” are beyond the reach of non‐specialists. In reality, many of these practices and skills are attainable, and we argue will need to become commonplace to meet One Health challenges.

The unique strength of long‐term research systems is then the ability to build linkages between these faunal dynamics and the existing robust local data streams that quantify environmental variance. For instance, the Konza LTER site in Kansas also supports long‐term sampling of biodiversity (including small mammals and ticks) through the National Ecological Observatory Network (NEON) that might subsequently be analyzed in conjunction with abiotic data collected through both LTER and NEON and disseminated through the Environmental Data Initiative (EDI). Additionally, the NEON aerial observation network allows us to track with fine‐scale resolution where grassland and woodland habitats come into contact (Figure 4). However, a lack of systematic sample collection of small mammals and other components of biodiversity necessary for rigorous epidemiological surveillance still impedes progress through these initiatives from the perspective of One Data and One Health (Cook et al. 2016).

The necessary prerequisites for robust One Health surveillance associated with long‐term ecological research could be realized through development of specimen archives consisting of site‐intensive targeted whole‐specimen sampling from long‐term research sites (Hope et al. 2018; Colella et al. 2021). Small mammals constitute the leading source of zoonoses globally, and generally these species are extremely resilient to inter‐annual population fluctuations. Modest specimen collection has been shown to have negligible impact on natural population trajectories (Hope et al. 2018). We have similarly found no detectable population impacts from nine consecutive years of specimen sampling from the Konza experimental system (data not yet published). Rodents within the families Cricetidae and Muridae combined constitute a quarter of all mammal species globally (~1600 species in these two families), contain the vast majority of known reservoirs of disease, and are generally common (< 10% of species recognized as endangered; IUCN 2024). These mammals essentially constitute a renewable resource from which the global scientific community might better understand more consequential human‐induced ecosystem perturbations and human health risks. Successful conservation efforts, including of small mammals, also rely heavily on resources within biorepositories, as declines in most species normally have no single cause that can be mitigated through monitoring and management of wild populations alone (Miller et al. 2020).

A major finding of our synthesis is a persistent long‐term decline in the deer mouse, one of the most common species of small mammal in North America, likely associated either directly or indirectly with an increase in woodland species. We argue that conservation of such common species, here associated with grasslands of the Great Plains, should be an urgent goal now, before they become more rare. This species likely plays a critical role in curtailing the spread of disease as part of the native grassland community, while it is also recognized as a model organism globally for the study of disease transmission (Barbour 2017). As deer mice decline, the recent increases in both white‐footed mice and hispid cotton rats ( *Sigmodon hispidus* ) through the Great Plains have concerning implications for further emergence of disease. Whereas these species have been afforded comparatively narrow focus for epidemiological exploration, major changes are happening now, and the resources we need to generate a holistic perspective on their role in disease emergence are as yet underrepresented (Thompson et al. 2021).

### 4.3 Current Limitations

The inferences we have made for disease risk across the Great Plains need much greater quantification through rigorous epidemiological investigation. Identification of ectoparasitic vectors and tracking of multi‐year trends of vector species and host‐vector relationships are in progress. Development of metagenomic surveillance of pathogens is also a critical next step, particularly through sequence‐based methods such as with emerging nanopore workflows (e.g., Kipp et al. 2023). This would enhance our ability to study prevalence, specificity, and phylogenetic relationships among pathogens and guilds of hosts. From a One Data perspective, our data were sourced from an experimental system where we mined data that would not be intuitively linked from the perspective of the broader scientific community. The critical need to advance One Health as presented here is for mobilization of the necessary relational linkages between the vast and growing data repositories that fuel still largely independent research priorities.

## 5 Conclusions

Understanding how ecosystems function is a primary goal of long‐term ecological research programs. While the impacts of an emergent disease are both acute and urgent from a One Health perspective, the processes of environmental change that enable shifts in relationships between pathogens, reservoirs, and other potential hosts (including humans) are more protracted and require often many years of data to resolve. If we can effectively mobilize relational linkages between digitized data streams from ecological, evolutionary, and biomedical components of One Health research, we might realize substantial benefits from embracing long‐term experimentally controlled systems to readily access information from across decadal timeframes. Equally, reciprocal data yielded from analyzes of small mammals, parasites, vectors, and other components of biodiversity might fill knowledge gaps towards recognizing critical linkages within complex ecological systems.

Conservation of grassland biodiversity across the Great Plains and other grassland systems globally may be the single biggest solution to reducing threats of emerging pathogens in such systems. However, mitigating native biodiversity declines across this region will require reducing the intensity of biotic interactions associated with encroaching woody plant communities and the breakdown of biodiversity filters that are otherwise enforced by larger tracts of intact native grasslands. The legacy of encroachment on small mammal communities is clearly long‐lasting, with remediation a difficult process. Additionally, the ongoing process of encroachment is contingent on both local and global forcing. Controlling for local ecological variables while studying the evolutionary dynamics of host–parasite interactions, all through integration of a One Data approach will provide valuable insight to the relative influence of local factors, versus global environmental trajectories, on human health outcomes. Our synthesis underscores this argument: local‐scale data built the mechanistic case that management can cause landcover change that enables increased contact between the faunal components that influence pathogen ecology, evolution, and subsequent disease dynamics. The data structure now exists for focusing big‐data investigations of these dynamics locally, regionally, and within other systems globally. But for practitioners to do so effectively will require expansion of both the purview of relational data linkages and standardized collection and preservation of the required biological source material.

## Author Contributions

**Andrew G. Hope:** conceptualization (lead), data curation (equal), formal analysis (equal), funding acquisition (lead), investigation (lead), methodology (lead), project administration (lead), writing – original draft (lead), writing – review and editing (equal). **Sam C. Speck:** conceptualization (supporting), data curation (equal), formal analysis (equal), investigation (equal), methodology (equal), writing – review and editing (equal). **Zak Ratajczak:** conceptualization (supporting), data curation (equal), formal analysis (equal), investigation (equal), methodology (equal), writing – review and editing (equal).

## Funding

This work was supported by the Directorate for Biological Sciences (Grants NSF2025849 and NSF2226917).

## Conflicts of Interest

The authors declare no conflicts of interest.

## Supporting information


**Figure S1:** Small mammal sampling of the three focal species from across the state of Kansas between 1980 and 2020. Abundances represent numbers of voucher specimens collected by year and archived in online digitized museum data aggregators (retrieved from GBIF.org on 23 September 2024). Trend lines show moving average abundance and a long‐term turnover in dominant species in the mid‐2000's. Green dashed line: Western deer mouse (*Peromyscus sonoriensis*); Orange dashed line: Hispid cotton rat ( *Sigmodon hispidus* ); Brown solid line: White‐footed mouse ( *Peromyscus leucopus* ).

**Data S1:** ece372982‐sup‐0002‐TableS1.pdf.

## Data Availability

Sequence data for specimens included in genetic analyzes are available through GenBank, https://www.ncbi.nlm.nih.gov/genbank/, and all accession numbers and associated museum specimen data are provided in the online Supporting Information (Table S1).
