# Supplementary figures and images for: Leveraging Long‐Term Ecological Research Initiatives Into the One Health Synthesis
Source: Ecol Evol. 2026 Jan 20;16(1):e72982. doi: 10.1002/ece3.72982 (PMC12819582; doi:10.1002/ece3.72982)

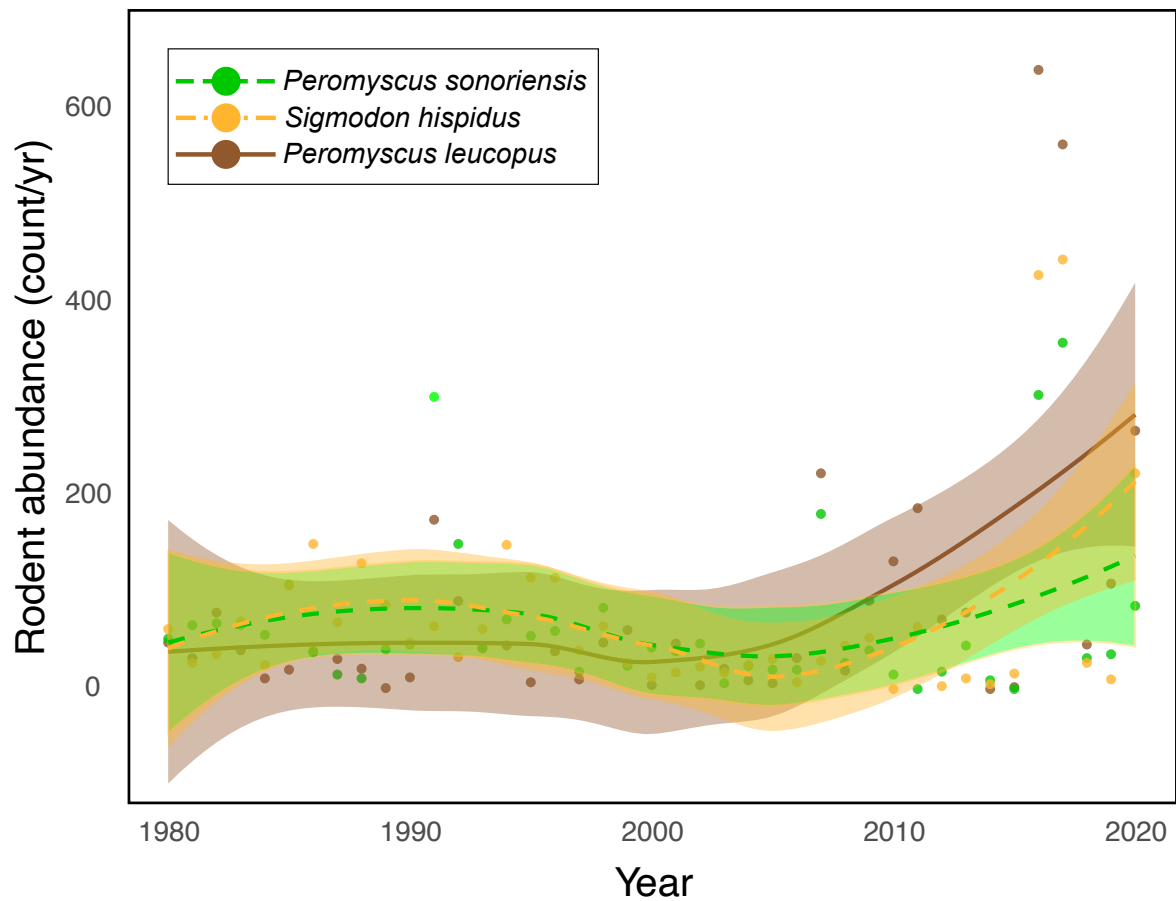

Supplement: Supplementary file 1 — Figure S1: Small mammal sampling of the three focal species from across the state of Kansas between 1980 and 2020. Abundances represent numbers of voucher specimens collected by year and archived in online digitized museum data aggregators (retrieved from GBIF.org on 23 September 2024). Trend lines show moving average abundance and a long‐term turnover in dominant species in the mid‐2000's. Green dashed line: Western deer mouse (Peromyscus sonoriensis); Orange dashed line: Hispid cotton rat ( Sigmodon hispidus ); Brown solid line: White‐footed mouse ( Peromyscus leucopus ). [file ECE3-16-e72982-s002.pdf]
