# Supplementary material for: Leveraging Long‐Term Ecological Research Initiatives Into the One Health Synthesis
Source: Ecol Evol. 2026 Jan 20;16(1):e72982. doi: 10.1002/ece3.72982 (PMC12819582; doi:10.1002/ece3.72982)
Supplement: Supplementary file 2 — Data S1: ece372982‐sup‐0002‐TableS1.pdf. [file ECE3-16-e72982-s001.pdf]

Table S1. Specimens examined for phylogenetic analysis of mtDNA cytochrome b gene sequences.

Species	Museum catalog number	Tissue Number	Country	State/Province	County	Specific Locality	Latitude	Longitude	Lineage	GenBank Acc.
Peromyscus leucopus	NA	NA	USA	Texas	NA	NA	NA	NA	South	AY041198
Peromyscus leucopus	NA	NA	Mexico	San Luis Potosi	NA	NA	NA	NA	South	AY859474
Peromyscus leucopus	NA	NA	USA	Illinois	NA	NA	NA	NA	North	BK010700
Peromyscus leucopus	NA	NA	USA	Texas	LaSalle	NA	NA	NA	South	DQ973104
Peromyscus leucopus	NA	NA	Canada	Ontario	Toronto	NA	NA	NA	North	EF989979
Peromyscus leucopus	NA	NA	Mexico	Tamaulipas	NA	NA	NA	NA	South	KJ810666
**Peromyscus leucopus**	**KSB:Mamm:686**	**KT0176**	**USA**	**Kansas**	**Harper County**	**Isabell Wildlife Area**	**37.499491**	**-98.551348**	**South**	**PV700426**
**Peromyscus leucopus**	**KSB:Mamm:142**	**KT0346**	**USA**	**Kansas**	**Ford County**	**No specific locality recorded.**	**37.73451**	**-98.56347**	**South**	**PV690518**
**Peromyscus leucopus**	**KSB:Mamm:793**	**KT0377**	**USA**	**Kansas**	**Chautauqua County**	**No specific locality recorded.**	**37.24833**	**-96.20165**	**Central**	**PV700427**
**Peromyscus leucopus**	**KSB:Mamm:133**	**KT0615**	**USA**	**Kansas**	**Miami County**	**La Cygne Wildlife Management Area**	**38.3988**	**-94.65171**	**North**	**PV690519**
**Peromyscus leucopus**	**KSB:Mamm:139**	**KT0650**	**USA**	**Kansas**	**Linn County**	**Marias des Cygnes**	**38.25348**	**-94.68842**	**Central**	**PV690520**
**Peromyscus leucopus**	**KSB:Mamm:140**	**KT0651**	**USA**	**Kansas**	**Linn County**	**Marias des Cygnes Wildlife Management Area, Plot 3-RG2-003**	**38.25357**	**-94.68863**	**Central**	**PV690521**
**Peromyscus leucopus**	**KSB:Mamm:736**	**KT0665**	**USA**	**Kansas**	**Shawnee County**	**Shawnee State Fishing Lake**	**39.209755**	**-95.802607**	**Central**	**PV690522**
**Peromyscus leucopus**	**KSB:Mamm:788**	**KT0869**	**USA**	**Ohio**	**Greene County**	**Indian Mound**	**39.73819**	**-83.82492**	**East**	**PV700428**
**Peromyscus leucopus**	**KSB:Mamm:1394**	**KT0963**	**USA**	**Kansas**	**Riley County**	**Konza Prairie Biological Station, Plot 20B**	**39.07453**	**-96.580085**	**Central**	**PV690523**
**Peromyscus leucopus**	**KSB:Mamm:1492**	**KT0965**	**USA**	**Kansas**	**Riley County**	**Konza Prairie Biological Station, Plot 20B**	**39.07348098**	**-96.57905898**	**North**	**PV690524**
**Peromyscus leucopus**	**KSB:Mamm:1443**	**KT0983**	**USA**	**Kansas**	**Riley County**	**Konza Prairie Biological Station, Plot 4B**	**39.07505804**	**-96.59674304**	**North**	**PV690525**
**Peromyscus leucopus**	**KSB:Mamm:1220**	**KT1074**	**USA**	**Kansas**	**Riley County**	**Konza Prairie Biological Station, Plot 20B**	**39.0740498**	**-96.58958296**	**North**	**PV690526**
**Peromyscus leucopus**	**KSB:Mamm:1409**	**KT1078**	**USA**	**Ohio**	**Greene County**	**Indian Mound**	**39.73875**	**-83.81384**	**East**	**PV690527**
**Peromyscus leucopus**	**KSB:Mamm:1466**	**KT1083**	**USA**	**Kansas**	**Osage County**	**Vassar**	**38.60981**	**-95.58775**	**North**	**PV690528**
**Peromyscus leucopus**	**KSB:Mamm:1410**	**KT1085**	**USA**	**Kansas**	**Osage County**	**Vassar**	**38.60996**	**-95.58736**	**North**	**PV700429**
**Peromyscus leucopus**	**KSB:Mamm:1850**	**KT2001**	**USA**	**Kansas**	**Cherokee County**	**Mined Lands Wildlife Management Area**	**37.20866**	**-95.00424**	**Central**	**PV690529**
**Peromyscus leucopus**	**KSB:Mamm:1852**	**KT2004**	**USA**	**Kansas**	**Cherokee County**	**Mined Lands Wildlife Management Area**	**37.20866**	**-95.00424**	**North**	**PV700430**
Peromyscus leucopus	NA	NA	USA	Virginia	NA	NA	NA	NA	East	KX784130
Peromyscus leucopus	NA	NA	USA	Rhode Island	NA	NA	NA	NA	East	KX784131
Peromyscus leucopus	NA	NA	USA	Rhode Island	NA	NA	NA	NA	East	KX784133
Peromyscus leucopus	NA	NA	USA	Massachusettes	NA	NA	NA	NA	East	KX784136
Peromyscus leucopus	NA	NA	Canada	Montreal	NA	NA	NA	NA	East	KX784138
Peromyscus leucopus	NA	NA	Canada	Montreal	NA	NA	NA	NA	East	KX784139
Peromyscus leucopus	NA	NA	Canada	Montreal	NA	NA	NA	NA	East	KX784140
Peromyscus leucopus	NA	NA	Canada	Montreal	NA	NA	NA	NA	East	KX784143
Peromyscus leucopus	NA	NA	Canada	Montreal	NA	NA	NA	NA	East	KX784144
Peromyscus leucopus	NA	NA	Canada	Montreal	NA	NA	NA	NA	East	KX784145
Peromyscus leucopus	NA	NA	Canada	Montreal	NA	NA	NA	NA	East	KX784147
Peromyscus leucopus	NA	NA	Canada	Montreal	NA	NA	NA	NA	East	KX784149
Peromyscus leucopus	NA	NA	Canada	Montreal	NA	NA	NA	NA	North	KX784154
Peromyscus leucopus	NA	NA	Canada	Montreal	NA	NA	NA	NA	North	KX784157
Peromyscus leucopus	NA	NA	Canada	Montreal	NA	NA	NA	NA	North	KX784159
Peromyscus leucopus	NA	NA	Canada	Montreal	NA	NA	NA	NA	North	KX784160
Peromyscus leucopus	NA	NA	Canada	Montreal	NA	NA	NA	NA	North	KX784161
Peromyscus leucopus	NA	NA	Canada	Montreal	NA	NA	NA	NA	North	KX784162
Peromyscus leucopus	NA	NA	Canada	Montreal	NA	NA	NA	NA	North	KX784163
Peromyscus leucopus	NA	NA	Canada	Montreal	NA	NA	NA	NA	East	KX784166
Peromyscus leucopus	NA	NA	USA	Texas	NA	NA	NA	NA	South	KY064165
Peromyscus leucopus	NA	NA	USA	Texas	NA	NA	NA	NA	South	KY064168
Peromyscus leucopus	NA	NA	USA	Oklahoma	NA	NA	NA	NA	Central	KY754106
Peromyscus leucopus	NA	NA	Mexico	LaPaz	NA	NA	NA	NA	South	MF589853
Peromyscus leucopus	NA	NA	USA	Illinois	NA	NA	NA	NA	North	MG674646
Peromyscus leucopus	NA	NA	USA	North Carolina	NA	NA	NA	NA	East	MG674647
Peromyscus leucopus	NA	NA	USA	New York	NA	NA	NA	NA	East	MG674648
Peromyscus leucopus	NA	NA	USA	Connecticut	NA	NA	NA	NA	East	MH256659
Peromyscus leucopus	NA	NA	USA	Michigan	NA	NA	NA	NA	East	MK410314
Peromyscus leucopus	NA	NA	USA	Missouri	NA	NA	NA	NA	North	MN124383
**Peromyscus leucopus**	**MSB:Mamm:331800**	**NK296346**	**USA**	**Kansas**	**Osage County**	**near intersection of W Booth and S Hoch Roads**	**38.50835**	**-95.86148**	**Central**	**PV700431**
**Peromyscus leucopus**	**MSB:Mamm:332417**	**NK305068**	**Canada**	**Manitoba**		**Fort Ellice Property (Nature Conservancy of Canada)**	**49.17253**	**-96.67281**	**North**	**PV700432**
**Peromyscus leucopus**	**MSB:Mamm:345521**	**NK305999**	**USA**	**Kansas**	**Scott County**	**Scott Lake Wildlife Management Area**	**38.68682**	**-100.93713**	**North**	**PV700433**

Peromyscus leucopus	MSB:Mamm:345687	NK306036	USA	Kansas	Scott County	Scott Lake Wildlife Management Area	38.68717	-100.93806	North	PV700434
Peromyscus leucopus	MSB:Mamm:345806	NK306136	USA	Kansas	Morton County	Cimarron National Grassland, Cimarron Recreation Area	37.13915	-101.82333	South	PV700435
Peromyscus leucopus	MSB:Mamm:345467	NK306178	USA	Kansas	Morton County	Cimarron National Grassland, Cottonwood Park	37.11238	-101.92808	South	PV700436
Peromyscus leucopus	MSB:Mamm:345605	NK306193	USA	Kansas	Jewell County	Lovewell Wildlife Area, Oak Hill Recreation Area	39.89455	-98.07394	North	PV700437
Peromyscus leucopus	MSB:Mamm:345836	NK306194	USA	Kansas	Jewell County	Lovewell Wildlife Area, Oak Hill Recreation Area	39.8942	-98.07378	North	PV700438
Peromyscus leucopus	MSB:Mamm:345669	NK306205	USA	Kansas	Jewell County	Lovewell Wildlife Area, Oak Hill Recreation Area	39.89268	-98.0731	North	PV700439
Peromyscus leucopus	MSB:Mamm:345516	NK306250	USA	Kansas	Republic County	Jamestown Wildlife Area	39.69545	-97.92265	North	PV700440
Peromyscus leucopus	MSB:Mamm:346050	NK306543	USA	Kansas	Barton County	Cheyenne Bottoms Wildlife Management Area	38.4757	-98.66773	Central	PV700441
Peromyscus leucopus	MSB:Mamm:346153	NK306544	USA	Kansas	Barton County	Cheyenne Bottoms Wildlife Management Area	38.44891	-98.75089	North	PV700442
Peromyscus leucopus	MSB:Mamm:346265	NK306564	USA	Kansas	Cherokee County	Mined Lands Wildlife Area	37.23057	-94.99105	North	PV700443
Peromyscus leucopus	MSB:Mamm:346285	NK306565	USA	Kansas	Cherokee County	Mined Lands Wildlife Area	37.2272	-94.98954	North	PV700444
Peromyscus leucopus	MSB:Mamm:346537	NK306709	USA	Kansas	Chautauqua County	Copan Wildlife Area	37.01767	-95.96779	Central	PV700445
Peromyscus leucopus	MSB:Mamm:346506	NK306713	USA	Kansas	Montgomery County	Elk City Wildlife Area	37.2448	-95.88008	North	PV700446
Peromyscus leucopus	MSB:Mamm:346549	NK306737	USA	Kansas	Chautauqua County	Copan Wildlife Area	37.01759	-95.96796	North	PV700447
Peromyscus leucopus	MSB:Mamm:347226	NK307100	USA	Kansas	Clark County	Clark State Fishing Lake and Wildlife Area	37.38864	-99.7789	South	PV700448
Peromyscus leucopus	MSB:Mamm:347135	NK307128	USA	Kansas	Ford County	Ford State Fishing Lake and Wildlife Area	37.81508	-99.91959	South	PV700449
Peromyscus leucopus	MSB:Mamm:347740	NK307218	USA	Missouri	Harrison County	31945 East 237 Street	40.37074	-93.821989	North	PV700450
Peromyscus leucopus	MSB:Mamm:347808	NK307233	USA	Missouri	Harrison County	31945 East 237 Street	40.37365	-93.8248	North	PV700451
